# Cadonilimab in combination with chemotherapy for HER2-negative advanced gastric or gastroesophageal junction adenocarcinoma: a cost-effectiveness analysis

**DOI:** 10.3389/fphar.2025.1646818

**Published:** 2025-07-29

**Authors:** Zuojuan Xiang, Wei Li, Qiao Xia

**Affiliations:** Department of Pharmacy, The Affiliated Cancer Hospital of Xiangya School of Medicine, Central South University, Hunan Cancer Hospital, Changsha, China

**Keywords:** cadonilimab, partitioned survival model, cost-effectiveness, PD-L1 expression, gastric or gastroesophageal junction adenocarcinoma

## Abstract

**Background:**

The COMPASSION-15 trial confirmed the safety and effectiveness of cadonilimab, a bispecific antibody targeting both programmed death 1 (PD-1) and cytotoxic T lymphocyte antigen-4 (CTLA-4), in treating human epidermal growth factor receptor 2 (HER2) negative advanced gastric or gastroesophageal junction adenocarcinoma (G/GEJA). Notably, it demonstrated significant survival benefits even in the low programmed death ligand 1 (PD-L1) expression subgroup, overcoming the limitations of current immunotherapy. This study aims to comprehensively evaluate its cost-effectiveness.

**Methods:**

The cost-effectiveness of cadonilimab plus chemotherapy compared to chemotherapy alone was evaluated using a partitioned survival model with a 10-year time horizon, based on data from the COMPASSION-15 trial. Incremental cost-effectiveness ratio (ICER) was estimated to ascertain the cost-effectiveness. Furthermore, subgroup analysis stratified by PD-L1 combined positive score (CPS) thresholds, as well as sensitivity and scenario analyses, were performed.

**Results:**

The estimated ICER value was $35,613.34/quality-adjusted life-year (QALY) for the entire cohort, $21,142.58/QALY for the high PD-L1 expression subgroup (CPS ≥5), and $45,000.62/QALY for the low PD-L1 expression subgroup (CPS <5). Only the high PD-L1 expression subgroup achieved the cost-effectiveness, as its ICER value was below the willingness-to-pay (WTP) threshold of $24,600/QALY. Sensitivity and scenario analyses demonstrated the robustness of the result.

**Conclusion:**

In China, incorporating cadonilimab with chemotherapy was found to be more cost-effective as a first-line treatment for HER2-negative advanced G/GEJA in the PD-L1 CPS ≥5 subgroup. Nevertheless, it was not cost-effective for either the entire cohort or the PD-L1 CPS <5 subgroup. These findings can provide valuable insights for future pricing strategies and healthcare decision-making.

## 1 Introduction

Gastric cancer is the fifth most prevalent cancer and the fifth leading cause of cancer-related mortality globally (Bray et al., 2024). There are significant regional variations in its incidence, with East Asia experiencing particularly high rates. In 2022, China reported 358,700 new cases and 260,400 deaths due to gastric cancer (Han et al., 2024). The lack of distinct early symptoms often results in diagnoses at advanced stages (Van Cutsem et al., 2016; Smyth et al., 2020). Recent therapeutic advancements for advanced gastric cancer have been made through the integration of immunotherapy and chemotherapy, particularly for patients with negative human epidermal growth factor receptor 2 (HER2) status and positive programmed death ligand 1 (PD-L1) expression. Phase 3 clinical trials have demonstrated that this combination therapy can prolong median overall survival (OS) by 3–5 months, thus establishing it as the first-line standard treatment. The immune checkpoint inhibitors (ICIs) utilized in this regimen include nivolumab, pembrolizumab, sintilimab, tislelizumab, and sugemalimab (Zhang et al., 2025; Janjigian et al., 2021; Rha et al., 2023; Xu et al., 2023; Qiu et al., 2024). Nonetheless, no programmed death 1 (PD-1)/PD-L1 inhibitor has yet demonstrated efficacy in patients with low PD-L1 expression. Therefore, new treatment options for these patients are urgently needed.

To improve patient response rates and survival outcomes, the combination of cytotoxic T lymphocyte antigen-4 (CTLA-4) and PD-1 blockers is advocated and has demonstrated synergistic anti-tumor effects in multiple solid tumors (Rotte, 2019; Larkin et al., 2015; Antonia et al., 2016; Overman et al., 2018). Cadonilimab, a bispecific antibody targeting both PD-1 and CTLA-4, has received approval for the treatment of cervical and gastric cancer in China (Keam, 2022; Pang et al., 2023). The phase 3 COMPASSION-15 study, conducted across 75 clinical sites in China, confirmed that incorporating cadonilimab with chemotherapy provided significant survival benefits for patients with HER2-negative advanced gastric or gastroesophageal junction adenocarcinoma (G/GEJA) as a first-line treatment, compared to chemotherapy alone (Shen et al., 2025). The study reported a median OS of 14.1 months *versus* 11.1 months in the entire cohort and 15.3 months *versus* 10.9 months in the subgroup with a PD-L1 combined positive score (CPS) ≥ 5. Importantly, there was also a notable improvement in the subgroup with a PD-L1 CPS <5, with a median OS of 13.7 months compared to 11.4 months. These findings indicated an unprecedented therapeutic efficacy in a patient subpopulation that has historically been difficult to treat.

It is noteworthy that, subsequent to price negotiations, cadonilimab has been incorporated into the 2025 Chinese medical insurance catalogue, with reimbursement specifically restricted to cervical cancer treatment. This addition significantly improves its accessibility and affordability. This study seeks to provide economic insights from the perspective of the Chinese healthcare system, thereby serving as a reference for optimizing healthcare resource allocation and rationalizing clinical drug use in the context of G/GEJA. The study adhered to the Consolidated Health Economic Evaluation Reporting Standards (CHEERS) reporting guideline (see Supplementary Table S1).

## 2 Methods

### 2.1 Patient and treatment

Patient baseline characteristics were obtained from the COMPASSION-15 trial cohort. Eligible participants were individuals with histologically confirmed unresectable, locally advanced, or metastatic G/GEJA who had not previously undergone systemic anticancer treatment. The age range of these participants was 18–75 years. Individuals with a known HER2-positive status were excluded from the study. Ethical approval was deemed unnecessary, as the study did not involve the recruitment of actual patients or the retrospective analysis of primary patient data.

Participants were randomly assigned to either the cadonilimab plus chemotherapy group or the chemotherapy group. Detailed information about the specific treatment regimens of both groups was provided in Table 1. After completing six therapy cycles, individuals in the cadonilimab plus chemotherapy group transitioned to a maintenance phase, continuing with cadonilimab at the same dosage until treatment discontinuation events occurred, such as disease progression, intolerable toxicity, or voluntary withdrawal. In contrast, the chemotherapy group did not receive maintenance therapy. The administration of cadonilimab was limited to a maximum duration of 2 years (Shen et al., 2025; Gauci et al., 2019; Borghaei et al., 2021).

**TABLE 1:** Treatment regimens.

Group	Dosing regimen	Maintenance therapy
Cado plus Chem	Cadonilimab was administered intravenously at a dosage of 10 mg/kg on the first day of every 3-week cycle, in combination with the XELOX regimen, for a total of six cycles	Cadonilimab was administered intravenously at a dosage of 10 mg/kg on the first day of every 3-week cycle
Chem	XELOX regimen for a total of six cycles	-

Cado, cadonilimab; Chem, chemotherapy; XELOX, oxaliplatin, administered intravenously at a dose of 130 mg/m^2^ on day 1, and capecitabine, administered orally at a dose of 1,000 mg/m^2^ twice daily from days 1–14, repeated every 3 weeks.

### 2.2 Model construction

**FIGURE 1:**
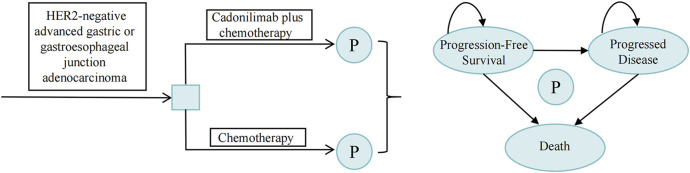
Schematic representation of the partitioned survival model. P, partitioned survival model.

A partitioned survival model was constructed using data from the COMPASSION-15 trial within TreeAge Pro 2022, incorporating three mutually exclusive health states: progression-free survival (PFS), progressive disease (PD), and death, as illustrated in Figure 1. The model was designed with a 10-year time horizon, at which point the mortality rate in each treatment arm exceeded 95%. The cycle length was set at 21 days, aligning with the dosing schedule of the COMPASSION-15 trial. The cost-effectiveness of the intervention was evaluated by comparing the incremental cost-effectiveness ratio (ICER) value to the predetermined willingness-to-pay (WTP) threshold. If the ICER value was below the WTP threshold, the intervention was considered cost-effective. The WTP threshold was set at 1.94 times the Gross Domestic Product (GDP) *per capita* in 2023 (Xu et al., 2024), equating to $24,600/quality-adjusted life-year (QALY).

To extract data points from survival curves for both the entire cohort and subgroups with high PD-L1 expression (CPS ≥5) as well as low PD-L1 expression (CPS <5), WebPlotDigitizer was employed. Subsequently, patient-level data were reconstructed using R (version 4.3.2). A range of parametric survival functions (Exponential, Weibull, Gamma, Log-normal, Log-logistic, Gompertz, and Generalized gamma) were utilized to extend survival curves beyond the clinical trial’s observation period (Guyot et al., 2012). The distribution with the lowest Akaike Information Criterion (AIC) and Bayesian Information Criterion (BIC) values, alongside visual inspection, was deemed the optimal distribution (Supplementary Table S2; Supplementary Figures S1-S3). Furthermore, the simulated survival curves were validated internally with COMPASSION-15 data and externally with CheckMate 649 and ORIENT-16 trial data. Table 2 presented the optimal distributions and key parameters.

**TABLE 2:** Key model input.

Parameters	Baseline value	Minimum	Maximum	Distribution	Source
Survival curves for overall population					
OS curve of Cado plus Chem arm	meanlog = 2.7400, sdlog = 0.9694			Log-normal	
OS curve of Chem arm	shape = 2.0250, scale = 11.449			Log-logistic	
PFS curve of Cado plus Chem arm	meanlog = 2.0967, sdlog = 0.9578			Log-normal	
PFS curve of Chem arm	shape = 2.1550, scale = 5.0930			Log-logistic	
Survival curves for PD-L1 CPS ≥ 5subgroup					
OS curve of Cado plus Chem arm	meanlog = 2.8510, sdlog = 1.1480			Log-normal	
OS curve of Chem arm	shape = 1.8481, rate = 0.1381			Gamma	
PFS curve of Cado plus Chem arm	meanlog = 2.1347, sdlog = 1.0133			Log-normal	
PFS curve of Chem arm	shape = 2.2200, rate = 0.3550			Gamma	
Survival curves for PD-L1 CPS < 5subgroup					
OS curve of Cado plus Chem arm	meanlog = 2.6391, sdlog = 0.9383			Log-normal	
OS curve of Chem arm	meanlog = 2.4292, sdlog = 0.8175			Log-normal	
PFS curve of Cado plus Chem arm	meanlog = 2.0431, sdlog = 0.9247			Log-normal	
PFS curve of Chem arm	shape = 2.0680, scale = 5.0710			Log-logistic	
COST ($)					
Radiological examination	171.03	136.82	205.24	Gamma	Lang et al. (2024)
Blood chemistry	24.69	19.75	29.63	Gamma	Lin et al. (2024)
Urinalysis	4.12	3.30	4.94	Gamma	Lin et al. (2024)
Coagulation function	9.13	7.30	10.96	Gamma	Lin et al. (2024)
Thyroid function	20.58	16.46	24.70	Gamma	Lin et al. (2024)
12-lead electrocardiogram	3.70	2.96	4.44	Gamma	(Lin et al., 2024)
Drug administration infusion	2.82	2.26	3.38	Gamma	Shen et al. (2022)
Best supportive care per cycle	164.57	131.66	197.48	Gamma	Lang et al. (2024)
Terminal care	1,460.30	1,168.24	1,752.36	Gamma	Lang et al. (2024)
Cost of drugs					
Cadonilimab/100 mg	211.16	168.93	253.39	Gamma	
Oxaliplatin/100 mg	62.19	49.75	74.63	Gamma	
Capecitabine/1000 mg	0.48	0.38	0.58	Gamma	
Paclitaxel/100 mg	17.01	13.61	20.41	Gamma	
Apatinib/100 mg	5.63	4.50	6.76	Gamma	
Cost of serious adverse events					
Decreased platelet count	1,522.49	1,217.99	1,826.98	Gamma	Chen et al. (2022)
Decreased neutrophil count	116.28	93.02	139.53	Gamma	Chen et al. (2022)
Decreased white blood cell count	473.01	378.41	567.61	Gamma	Liu et al. (2023)
Anemia	473.34	378.67	568.01	Gamma	Chen et al. (2022)
Hypokalemia	325.85	260.68	391.01	Gamma	Shao et al. (2022)
Utility					
PFS	0.80	0.64	0.96	Beta	Shiroiwa et al. (2011)
PD	0.58	0.46	0.69	Beta	Shiroiwa et al. (2011)
Disutility					
Decreased platelet count	0.11	0.09	0.13	Beta	Lang et al. (2024)
Decreased neutrophil count	0.20	0.16	0.24	Beta	Chen et al. (2022)
Decreased white blood cell count	0.20	0.16	0.24	Beta	Chen et al. (2022)
Anemia	0.07	0.06	0.08	Beta	Chen et al. (2022)
Hypokalemia	0.03	0.02	0.04	Beta	Shao et al. (2022)
Risk of serious AEs in Cado plus Chem group					
Decreased platelet count	0.29	0.23	0.34	Beta	Shen et al. (2025)
Decreased neutrophil count	0.15	0.12	0.18	Beta	Shen et al. (2025)
Decreased white blood cell count	0.07	0.06	0.09	Beta	Shen et al. (2025)
Anemia	0.10	0.08	0.12	Beta	Shen et al. (2025)
Hypokalemia	0.06	0.05	0.07	Beta	Shen et al. (2025)
Risk of serious AEs in Cado group					
Decreased platelet count	0.25	0.20	0.30	Beta	Shen et al. (2025)
Decreased neutrophil count	0.15	0.12	0.18	Beta	Shen et al. (2025)
Decreased white blood cell count	0.06	0.05	0.08	Beta	Shen et al. (2025)
Anemia	0.13	0.10	0.15	Beta	Shen et al. (2025)
Hypokalemia	0.01	0.01	0.01	Beta	Shen et al. (2025)
Body surface area, m^2^	1.72	1.38	2.06	Gamma	Shu et al. (2022)
Body weight, kg	65.00	52.00	78.00	Gamma	Shu et al. (2022)
Discount rate (%)	5.00	0.00	8.00	Fix	

Cado, cadonilimab; Chem, chemotherapy; OS, overall survival; PFS, progression-free survival; PD, progressive disease; PD-L1, programmed death ligand 1; CPS, combined positive score; AE, adverse event.

### 2.3 Cost and utility

As illustrated in Table 2, this study exclusively considered direct medical costs, encompassing laboratory tests, radiological examinations, medication expenditures, injection costs, best supportive care, end-of-life care, and cost associated with the management of adverse events (AEs). In accordance with the COMPASSION-15 protocol, patients underwent radiological examinations every 6 weeks during the initial 54 weeks, followed by examinations every 9 weeks thereafter. Additionally, 12-lead electrocardiograms, coagulation function tests, and thyroid function tests were conducted bi-cyclically. Given that oxaliplatin, capecitabine, and paclitaxel were included in China’s volume-based procurement program, their prices were established based on the winning bids. The prices of cadonilimab and apatinib were sourced from the database of Hunan Provincial Healthcare Security Administration. These prices could represent the latest drug prices at public hospitals in China. Based on the reported exposure times and medication dosages in the COMPASSION-15 study, we implemented corresponding adjustments in our model, presuming cadonilimab administration for eight cycles and other medications for 6 cycles. Patients were assumed to receive paclitaxel monotherapy following disease progression. Other cost data were extracted from existing literature. Due to the lack of health-related quality of life data in the COMPASSION-15 study, utility and disutility values associated with AEs were also obtained from the literature. The model concentrated on grade ≥3 AEs with an incidence rate exceeding 3% in any treatment group, assuming these events occurred during the initial cycle, as grade 1–2 AEs were generally manageable. All costs were converted to U.S. dollars using the 2023 exchange rate of 1 USD = 7.0467 RMB and adjusted to 2023 level using the Consumer Price Index. Additionally, cost and utility data were discounted annually at a rate of 5%.

### 2.4 Sensitivity analysis

To assess the robustness of the model, both one-way and probabilistic sensitivity analyses were performed. A one-way sensitivity analysis examined how changes in individual factors affected model outcomes, using parameter ranges from existing literature or adjusted by ±20% of the base-case value. Conversely, the probabilistic sensitivity analysis entailed the specification of distributions for each parameter, followed by the execution of Monte Carlo simulations with 1,000 iterations to evaluate the impact of concurrent changes in all parameters on the model outcomes. Table 2 contained detailed parameter inputs.

### 2.5 Scenario analysis

#### 2.5.1 Scenario 1

Drawing on the distribution of various second-line treatments reported in the COMPASSION-15 trial, we incorporated a more comprehensive range of second-line treatment options for this scenario, including chemotherapy, immunotherapy, and targeted therapy. Immunotherapy continued to be represented by cadonilimab, as demonstrated by the COMPASSION-15 trial which indicated that patients who prematurely discontinued cadonilimab due to pseudoprogression or isolated responses could still benefit from cadonilimab monotherapy, if it was well tolerated (Billan et al., 2020; Spagnolo et al., 2021). Detailed information regarding specific treatment dosages and proportions was provided in Supplementary Table S3.

#### 2.5.2 Scenario 2

In this scenario, the time horizon was set to 5 years to assess the influence of simulation duration on the outcomes.

#### 2.5.3 Scenario 3

Cadonilimab was exclusively available in a 125 mg formulation, and patient dosages were determined based on body weight, which could result in dose wastage. In this scenario,the economic evaluation model incorporates the extra cost of this wastage.

## 3 Results

### 3.1 Base-case analysis

As summarized in Table 3, the cadonilimab group achieved 1.16 QALYs at a total cost of $21,809.45, whereas the chemotherapy group achieved 0.80 QALYs at a total cost of $8,680.07. The corresponding ICER value was estimated to be $35,613.34/QALY, which surpassed the WTP threshold of $24,600/QALY. This indicated that the addition of cadonilimab to the treatment regimen did not constitute a cost-effective strategy.

**TABLE 3:** Results of base-case and scenario analyses.

Treatment	Total cost ($)	Incremental costs ($)	QALYs	Incermental QALYs	ICER ($/QALY)
Base-case analysis					
Overall population					
Chem	8,680.07		0.80		
Cado plus Chem	21,809.45	13,129.38	1.16	0.37	35,613.34
PD-L1 CPS ≥5					
Chem	7,676.28		0.64		
Cado plus Chem	23,332.42	15,656.13	1.38	0.74	21,142.58
PD-L1 CPS <5					
Chem	8,384.00		0.76		
Cado plus Chem	21,040.94	12,656.90	1.04	0.28	45,000.62
Scenario analysis 1					
Overall population	15,293.69		0.80		
Chem	28,018.71	12,725.03	1.16	0.37	34,516.54
Cado plus Chem					
PD-L1 CPS ≥5					
Chem	12,862.43		0.64		
Cado plus Chem	31,674.73	18,812.30	1.38	0.74	25,404.78
PD-L1 CPS <5					
Chem	14,283.40		0.76		
Cado plus Chem	26,411.74	12,128.34	1.04	0.28	43,121.35
Scenario analysis 2					
Overall population					
Chem	8,411.52		0.76		
Cado plus Chem	21,193.95	12,872.43	1.08	0.32	39,962.69
PD-L1 CPS ≥5					
Chem	7,672.66		0.64		
Cado plus Chem	22,053.12	14,380.46	1.21	0.57	25,269.12
PD-L1 CPS <5					
Chem	8,273.23		0.74		
Cado plus Chem	20,610.72	12,337.49	0.98	0.24	51,508.01
Scenario analysis 3					
Overall population					
Chem	8,680.07		0.80		
Cado plus Chem	23,503.6	14,823.53	1.16	0.37	40,208.70
PD-L1 CPS ≥5					
Chem	7,676.28		0.64		
Cado plus Chem	25,025.66	17,349.37	1.38	0.74	23,429.19
PD-L1 CPS <5					
Chem	8,384.04		0.76		
Cado plus Chem	22,735.35	14,351.31	1.04	0.28	51,024.96

Cado, cadonilimab; Chem, chemotherapy; QALYs, quality-adjusted life-years; ICER, incremental cost-effectiveness ratio; PD-L1, programmed death ligand 1.

Subgroup analysis indicated that in the high PD-L1 expression subgroup, the cadonilimab group gained 0.74 additional QALYs over the chemotherapy group, a figure notably higher than the additional 0.28 QALYs observed in the low PD-L1 expression subgroup. The corresponding incremental costs were $15,656.13 and $12,656.90, respectively. Consequently, the ICER for the high PD-L1 expression subgroup was $21,142.58/QALY, falling below the WTP threshold, whereas for the low PD-L1 expression subgroup, it was $45,000.62/QALY, exceeding the threshold.

### 3.2 Sensitivity analysis

**FIGURE 2:**
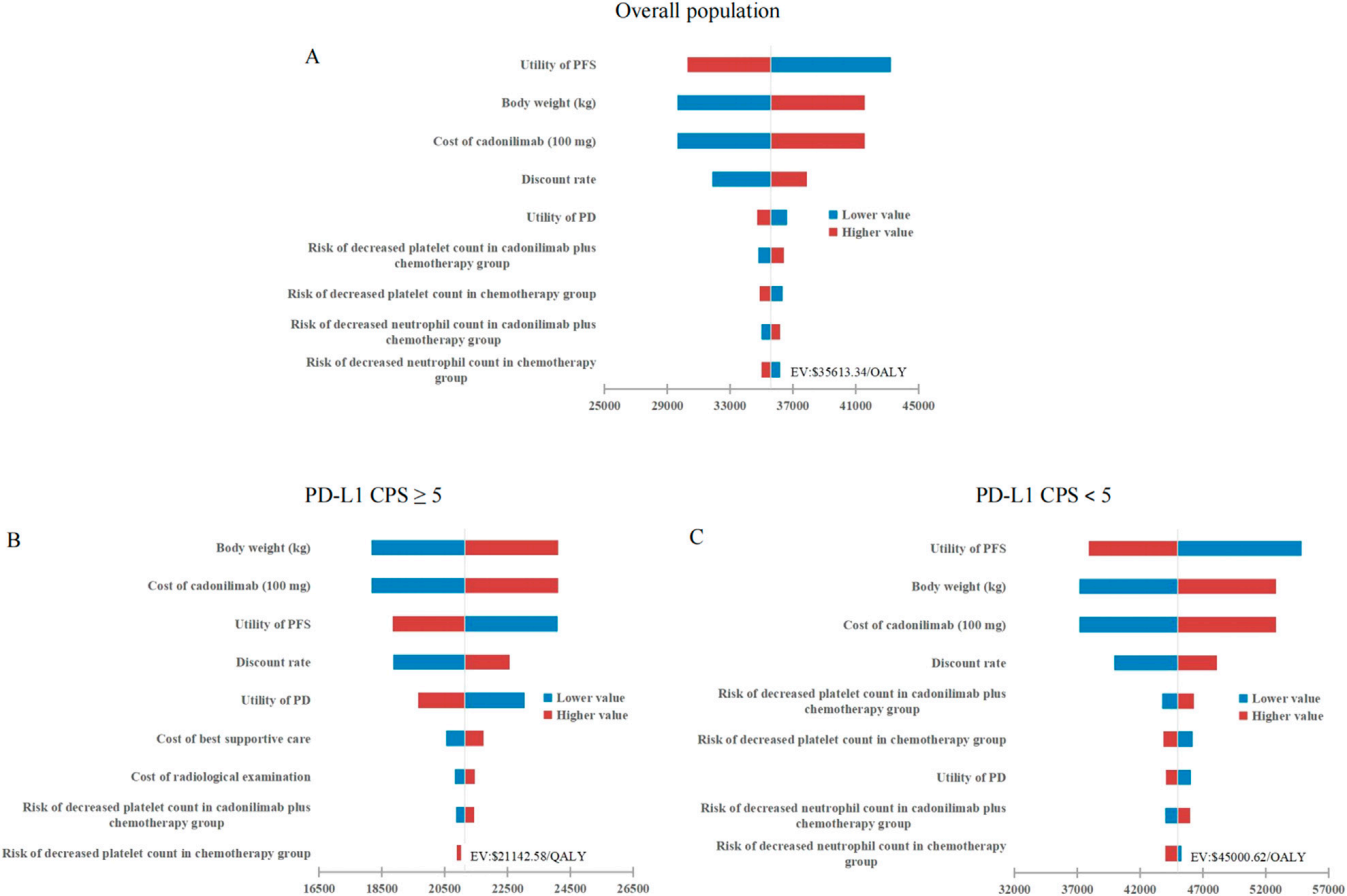
Tornado diagrams stratified by PD-L1 CPS thresholds. **(A)** Overall population. **(B)** High PD-L1 expression subgroup. **(C)** Low PD-L1 expression subgroup. PFS, progression-free survival; PD, progressed disease; QALY, quality-adjusted life-year; PD-L1 programmed death ligand 1; CPS, combined positive score; WTP, willingness-to-pay.

As depicted in Figure 2, tornado diagrams illustrated the top 10 factors exerting the most significant influence on the model. The model’s outcomes were primarily affected by the PFS utility, body weight, and the cost of cadonilimab. Nonetheless, none of these variables substantially altered the model results, underscoring the model’s robustness across the entire cohort and the two subgroups.

**FIGURE 3:**
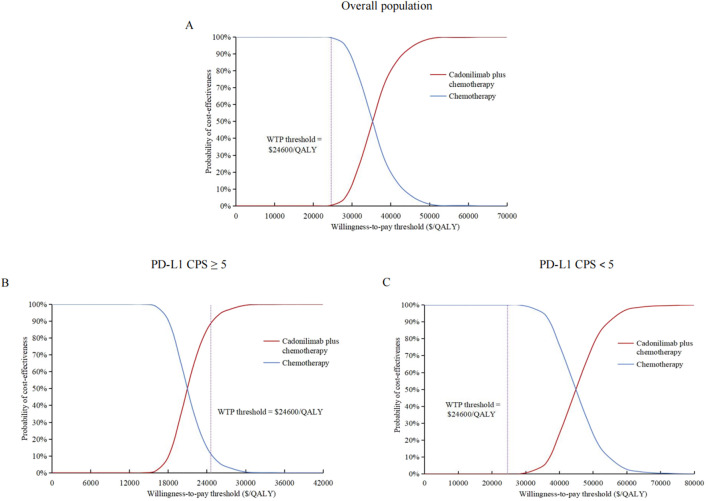
Cost-effectiveness acceptability curves. **(A)** Overall population. **(B)** High PD-L1 expression subgroup. **(C)** Low PD-L1 expression subgroup. QALY, quality-adjusted life-year; PD-L1 programmed death ligand 1; CPS, combined positive score; WTP, willingness-to-pay.

**FIGURE 4:**
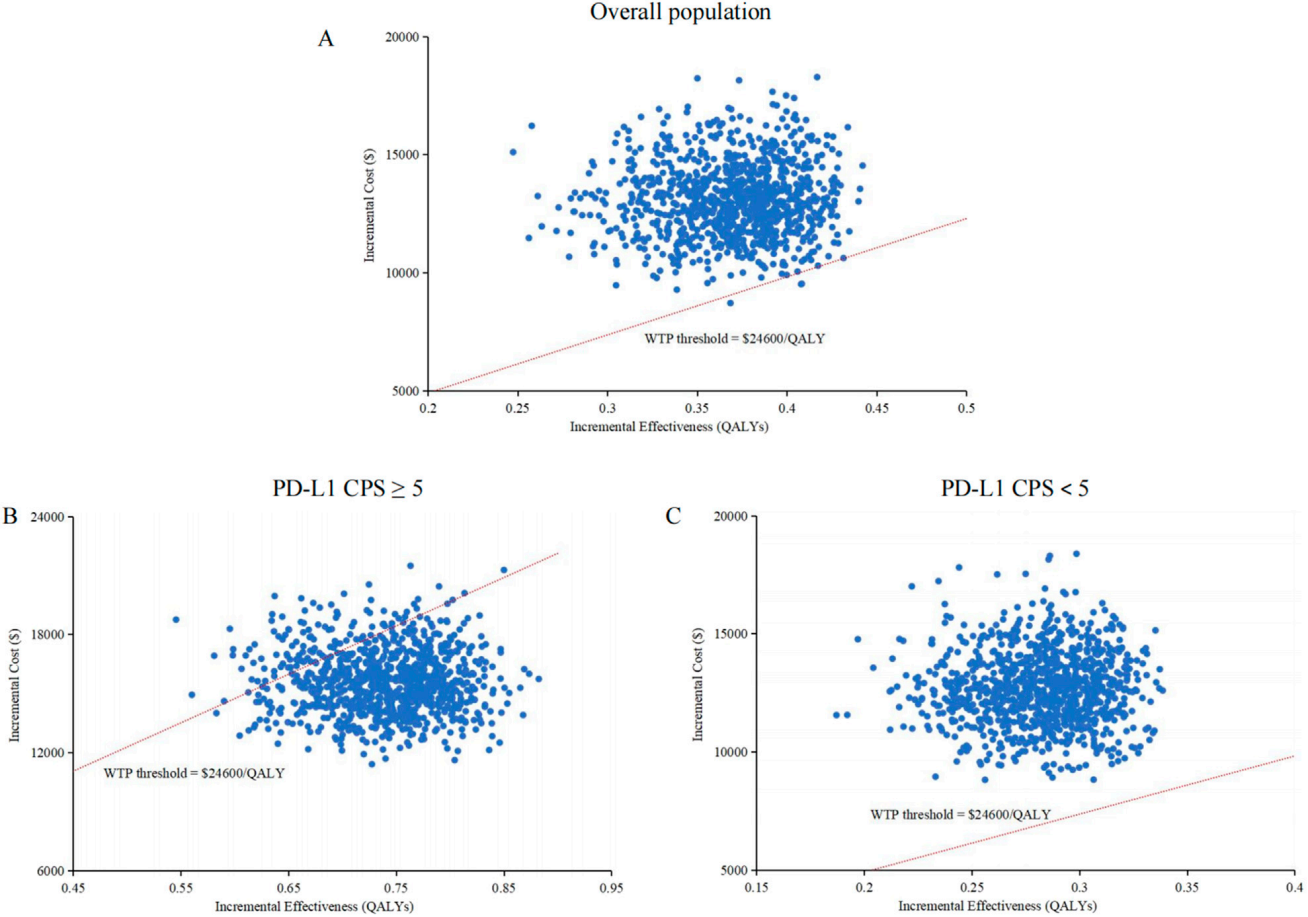
Scatter plots. **(A)** Overall population. **(B)** High PD-L1 expression subgroup. **(C)** Low PD-L1 expression subgroup. QALY, quality-adjusted life-year; PDL1 programmed death ligand 1; CPS, combined positive score; WTP, willingness-to-pay.

The cost-effectiveness acceptability curves indicated that the likelihood of cost-effectiveness for cadonilimab combination therapy increased with rising WTP threshold, as shown in Figure 3. At a WTP threshold of $24,600/QALY, the probability of cost-effectiveness for cadonilimab combination therapy in the entire cohort was 0.3%, in the high PD-L1 expression subgroup was 88.4%, and in the low PD-L1 expression subgroup was 0%, as displayed in the scatter plot in Figure 4.

### 3.3 Scenario analysis

The findings from the three scenario analyses aligned with those of the base-case analysis, indicating that the cadonilimab combination therapy was cost-effective within the high PD-L1 expression subgroup. Specifically, the ICER values for scenarios one to three were $25,404.78/QALY, $25,269.12/QALY, and $23,429.19/QALY, respectively, within this subgroup. In contrast, it did not exhibit economic advantages in the entire cohort, with ICER values of $34,516.54/QALY, $39,962.69/QALY, and $40,208.70/QALY, respectively, nor in the low PD-L1 expression subgroup, where the ICER values were $43,121.35/QALY, $51,508.01/QALY, and $51,024.96/QALY, respectively, as detailed in Table 3.

## 4 Discussion

In China, the proportion of gastric cancer patients with a PD-L1 CPS <5 is as high as 50.8% (Zhang et al., 2021), highlighting the exceptional significance of cadonilimab’s approval. However, this study indicated that cadonilimab combination therapy did not achieve cost-effectiveness in either this subgroup or the overall population, except for the subgroup with a PD-L1 CPS ≥5. The observed variation in outcomes across subgroups was primarily attributed to significant differences in the incremental effects among diverse patient populations, as illustrated in Table 3. Specifically, the incremental effect in the PD-L1 CPS ≥5 subgroup was more than twice that observed in the PD-L1 CPS <5 subgroup. This discrepancy was attributed to the fact that, although cadonilimab was a bispecific antibody, its clinical benefits were enhanced with higher levels of PD-L1 expression, consistent with its performance in other solid tumors, such as cervical cancer (Wu et al., 2024). The tornado diagram revealed that the PFS utility value was the most influential factor affecting the model outcomes for both the entire cohort and the PD-L1 CPS <5 subgroup. This finding indirectly suggested that inadequate clinical benefit might be a primary contributor to the loss of cost-effectiveness. Furthermore, the cost of cadonilimab emerged as a significant determinant impacting the results. To achieve cost-effectiveness, the price of cadonilimab would need to be reduced by an additional 36.87% for the overall population and by 52.10% for the PD-L1 CPS <5 subgroup. It should be noted that, according to the 2020 edition of the China Guidelines for Pharmacoeconomic Evaluations, an ICER value that is less than three times the GDP *per capita* signifies an acceptable level of cost increase. This study, however, adopted a more stringent WTP threshold in the context of terminal illnesses, specifically 1.94 times the GDP *per capita* in 2023. This threshold was informed by research conducted by Xu L. et al., which investigated WTP thresholds across various disease scenarios in China (Xu et al., 2024).

In contrast to the more costly options of nivolumab, pembrolizumab, and sugemalimab, both sintilimab and tislelizumab have been included in China’s medical insurance coverage for the treatment of gastric cancer. While sintilimab did not demonstrate an OS benefit in patients with a PD-L1 CPS <5, our previous research established its cost-effectiveness in China, even within this subgroup. This finding might be attributed to its comparatively lower price and notable improvement in PFS (Xiang et al., 2024). Similarly, tislelizumab showed cost-effectiveness in patients with positive PD-L1 expression; however, the phase 3 clinical trial did not encompass patients with negative PD-L1 expression, making it impossible to evaluate its cost-effectiveness in this subpopulation (Lang et al., 2024; Xu et al., 2024). Other economic evaluations indicated that nivolumab or pembrolizumab plus chemotherapy was not deemed cost-effective in either China or the United States (Shu et al., 2022; Zheng et al., 2024; Lang et al., 2024; Lang et al., 2023; Zhang et al., 2023; Marupuru et al., 2023; Cao et al., 2023). These findings indicated that cadonilimab might exhibit a more favorable cost-effectiveness profile relative to high-cost drugs such as nivolumab, pembrolizumab, and sugemalimab. Nonetheless, its economic advantages might be less pronounced when compared to more affordable alternatives like sintilimab. It was important to acknowledge that these pharmacoeconomic evaluations did not account for the costs associated with testing for PD-L1 expression levels. Cadonilimab eliminates the necessity for PD-L1 testing, thereby conserving testing resources, reducing associated costs, and minimizing delays in diagnosis and treatment, which provides a comparative advantage over other ICIs. Additionally, domestically produced ICIs in China have shown superior efficacy compared to imported counterparts and have been incorporated into the national medical insurance system. This integration has significantly alleviated the financial burden of disease and improved access to medications. Such developments have played a pivotal role in advancing the standardization of medical practices in China. Given that bispecific antibodies have addressed the limitations of traditional ICIs, if reimbursement for cadonilimab is broadened to encompass gastric cancer indications, it is expected that a larger number of patients will benefit from this treatment. Drugs included in the medical insurance list through price negotiations may undergo additional price or reimbursement adjustments during the annual update. Thus, our data can serve as valuable references for healthcare decision-making, such as determining whether gastric cancer indications should be included in medical insurance reimbursement and whether to consider PD-L1 expression levels, and also for manufacturers in devising future pricing strategies.

This study possesses two primary strengths. Firstly, the inclusion of exclusively Chinese patients in the COMPASSION-15 study mitigated potential biases arising from regional or population heterogeneity within the model. Secondly, the COMPASSION-15 study provided survival curves not only for the entire intention-to-treat population but also for two subgroups. As a result, a partitioned survival model, which utilizes survival curves by directly extracting survival data, was employed. This approach was likely to more accurately represent the actual survival outcomes of patients, thereby enhancing the reliability of the analysis.

There are also several limitations. Firstly, lack of direct comparative clinical data prevented a comparison between cadonilimab and other first-line standard ICIs, such as nivolumab, pembrolizumab, sintilimab, tislelizumab, and sugemalimab. Future economic evaluations of these drugs may be performed using a meta-analysis approach. Secondly, the extrapolation of survival data using parametric survival functions could affect the results. Despite the maturity of the survival data from COMPASSION-15 and the rigorous internal and external validation of the simulated curves, it remains essential to validate the model with long-term real-world data to enhance its reliability. Thirdly, utility values were derived from existing literature, potentially misrepresenting real-world data. However, the sensitivity analysis demonstrated that variations within the specified range did not impact the model’s outcomes. Fourthly, while the WTP threshold employed in this study was generally considered appropriate for China’s oncology sector based on existing evidence, it did not account for regional income disparities within China, which could affect the outcomes. Fifthly, in alignment with COMPASSION-15 trial, this pharmacoeconomic evaluation was conducted exclusively within China. It is anticipated that further validation of cadonilimab’s efficacy and safety will be undertaken in regions outside of China. In certain developed countries with higher WTP thresholds, the probability of cadonilimab achieving cost-effectiveness across diverse populations may be enhanced.

In conclusion, cadonilimab plus chemotherapy as a first-line treatment for HER2-negative advanced G/GEJA was found to be more cost-effective than chemotherapy alone in patients with a PD-L1 CPS ≥5. However, this combination therapy did not demonstrate cost-effectiveness in the overall population or in the subgroup with a PD-L1 CPS of <5. This study offers insights for optimizing healthcare resource allocation and developing evidence-based pricing strategies. A more comprehensive budget impact analysis is still required.

## Data Availability

The original contributions presented in the study are included in the article/Supplementary Material, further inquiries can be directed to the corresponding authors.

## References

[B1] Antonia S. J. López-Martin J. A. Bendell J. Ott P. A. Taylor M. Eder J. P. (2016). Nivolumab alone and nivolumab plus ipilimumab in recurrent small-cell lung cancer (checkMate 032): a multicentre, open-label, phase 1/2 trial. Lancet Oncol. 17 (7), 883–895. 10.1016/S1470-2045(16)30098-5 27269741

[B2] Billan S. Kaidar-Person O. Gil Z. (2020). Treatment after progression in the era of immunotherapy. Lancet Oncol. 21 (10), e463–e476. 10.1016/S1470-2045(20)30328-4 33002442

[B3] Borghaei H. Gettinger S. Vokes E. E. Chow L. Q. M. Burgio M. A. de Castro Carpeno J. (2021). Five-year outcomes from the randomized, phase III trials CheckMate 017 and 057: nivolumab *versus* docetaxel in previously treated non-small-cell lung cancer. J. Clin. Oncol. 39 (7), 723–733. 10.1200/JCO.20.01605 33449799 PMC8078445

[B4] Bray F. Laversanne M. Sung H. Ferlay J. Siegel R. L. Soerjomataram I. (2024). Global cancer statistics 2022: GLOBOCAN estimates of incidence and mortality worldwide for 36 cancers in 185 countries. CA Cancer J. Clin. 74 (3), 229–263. Epub 2024 Apr 4. 10.3322/caac.21834 38572751

[B5] Cao X. Zhang M. Li N. Zheng B. Liu M. Song X. (2023). First-line nivolumab plus chemotherapy *versus* chemotherapy alone for advanced gastric cancer, gastroesophageal junction cancer, and esophageal adenocarcinoma: a cost-effectiveness analysis. Ther. Adv. Med. Oncol. 15, 17588359231171038. 10.1177/17588359231171038 37223263 PMC10201153

[B6] Chen P. Li Y. Jing X. Chen J. Chen S. Yang Q. (2022). Cost-effectiveness analysis of sugemalimab in combination with chemotherapy as first-line treatment in Chinese patients with metastatic NSCLC. Lung Cancer 174, 157–164. 10.1016/j.lungcan.2022.11.008 36413882

[B7] Gauci M. L. Lanoy E. Champiat S. Caramella C. Ammari S. Aspeslagh S. (2019). Long-term survival in patients responding to anti-PD-1/PD-L1 therapy and disease outcome upon treatment discontinuation. Clin. Cancer Res. 25 (3), 946–956. 10.1158/1078-0432.CCR-18-0793 30297458

[B8] Guyot P. Ades A. E. Ouwens M. J. Welton N. J. (2012). Enhanced secondary analysis of survival data: reconstructing the data from published kaplan-meier survival curves. BMC Med. Res. Methodol. 12, 9–13. 10.1186/1471-2288-12-9 22297116 PMC3313891

[B9] Han B. Zheng R. Zeng H. Wang S. Sun K. Chen R. (2024). Cancer incidence and mortality in China, 2022. J. Natl. Cancer Cent. 4 (1), 47–53. 10.1016/j.jncc.2024.01.006 39036382 PMC11256708

[B10] Janjigian Y. Y. Shitara K. Moehler M. Garrido M. Salman P. Shen L. (2021). First-line nivolumab plus chemotherapy *versus* chemotherapy alone for advanced gastric, gastro-oesophageal junction, and oesophageal adenocarcinoma (checkMate 649): a randomised, open-label, phase 3 trial. Lancet 398 (10294), 27–40. 10.1016/S0140-6736(21)00797-2 34102137 PMC8436782

[B11] Keam S. J. (2022). Cadonilimab: first approval. Drugs 82 (12), 1333–1339. 10.1007/s40265-022-01761-9 35986837

[B12] Lang W. Ai Q. Zhang W. Jiang Q. He Y. Ouyang M. (2024). Cost-effectiveness analysis of tislelizumab plus chemotherapy *versus* placebo plus chemotherapy as first-line treatment for advanced gastric or gastroesophageal junction adenocarcinoma: perspectives from the United States and China. Front. Pharmacol. 15, 1461571. 10.3389/fphar.2024.1461571 39635432 PMC11614636

[B13] Lang W. Deng L. Lu M. Ouyang M. (2024). Cost-effectiveness analysis of pembrolizumab plus chemotherapy *versus* placebo plus chemotherapy for HER2-negative advanced gastric/gastroesophageal junction cancer in the Chinese healthcare system. Expert Rev. Pharmacoecon Outcomes Res. 24 (8), 1027–1042. 10.1080/14737167.2024.2378983 38979910

[B14] Lang Y. Lin Y. Li D. Liu J. Liu X. (2023). Pembrolizumab alone or in combination with chemotherapy *versus* chemotherapy for advanced gastric cancer: a cost-effectiveness analysis. Cancer Med. 12 (18), 18447–18459. 10.1002/cam4.6389 37706223 PMC10557869

[B15] Larkin J. Chiarion-Sileni V. Gonzalez R. Grob J. J. Cowey C. L. Lao C. D. (2015). Combined nivolumab and ipilimumab or monotherapy in untreated melanoma. N. Engl. J. Med. 373 (1), 23–34. 10.1056/NEJMoa1504030 26027431 PMC5698905

[B16] Lin Y. T. Wang C. He X. Y. Yao Q. M. Chen J. (2024). Comparative cost-effectiveness of first-line pembrolizumab plus chemotherapy vs. chemotherapy alone in persistent, recurrent, or metastatic cervical cancer. Front. Immunol. 14, 1345942. 10.3389/fimmu.2023.1345942 38274823 PMC10808689

[B17] Liu L. Wang L. Chen L. Ding Y. Zhang Q. Shu Y. (2023). Cost-effectiveness of sintilimab plus chemotherapy *versus* chemotherapy alone as first-line treatment of locally advanced or metastatic oesophageal squamous cell carcinoma. Front. Immunol. 14, 1092385. 10.3389/fimmu.2023.1092385 36756110 PMC9899904

[B18] Marupuru S. Arku D. Axon D. R. Villa-Zapata L. Yaghoubi M. Slack M. K. (2023). Cost-effectiveness analysis of nivolumab-chemotherapy as first-line therapy for locally advanced/metastatic gastric cancer: a United States payer perspective. Expert Rev. Pharmacoecon Outcomes Res. 23 (7), 831–841. 10.1080/14737167.2023.2219448 37243493

[B19] Overman M. J. Lonardi S. Wong K. Y. M. Lenz H. J. Gelsomino F. Aglietta M. (2018). Durable clinical benefit with nivolumab plus ipilimumab in DNA mismatch repair-deficient/microsatellite instability-high metastatic colorectal cancer. J. Clin. Oncol. 36 (8), 773–779. 10.1200/JCO.2017.76.9901 29355075

[B20] Pang X. Huang Z. Zhong T. Zhang P. Wang Z. M. Xia M. (2023). Cadonilimab, a tetravalent PD-1/CTLA-4 bispecific antibody with trans-binding and enhanced target binding avidity. MAbs 15 (1), 2180794. 10.1080/19420862.2023.2180794 36872527 PMC10012886

[B21] Qiu M. Z. Oh D. Y. Kato K. Arkenau T. Tabernero J. Correa M. C. (2024). Tislelizumab plus chemotherapy *versus* placebo plus chemotherapy as first line treatment for advanced gastric or gastro-oesophageal junction adenocarcinoma: RATIONALE-305 randomised, double blind, phase 3 trial. BMJ 385, e078876. 10.1136/bmj-2023-078876 38806195

[B22] Rha S. Y. Oh D. Y. Yañez P. Bai Y. Ryu M. H. Lee J. (2023). Pembrolizumab plus chemotherapy *versus* placebo plus chemotherapy for HER2-negative advanced gastric cancer (KEYNOTE-859): a multicentre, randomised, double-blind, phase 3 trial. Lancet Oncol. 24 (11), 1181–1195. 10.1016/S1470-2045(23)00515-6 37875143

[B23] Rotte A. (2019). Combination of CTLA-4 and PD-1 blockers for treatment of cancer. J. Exp. Clin. Cancer Res. 38 (1), 255. 10.1186/s13046-019-1259-z 31196207 PMC6567914

[B24] Shao T. Zhao M. Tang W. (2022). Cost-effectiveness analysis of sintilimab vs. placebo in combination with chemotherapy as first-line therapy for local advanced or metastatic oesophageal squamous cell carcinoma. Front. Oncol. 12, 953671. 10.3389/fonc.2022.953671 36561521 PMC9763586

[B25] Shen J. Du Y. Shao R. Jiang R. (2022). First-line sintilimab plus chemotherapy in locally advanced or metastatic esophageal squamous cell carcinoma: a cost-effectiveness analysis from China. Front. Pharmacol. 13, 967182. 10.3389/fphar.2022.967182 36569294 PMC9767976

[B26] Shen L. Zhang Y. Li Z. Zhang X. Gao X. Liu B. (2025). First-line cadonilimab plus chemotherapy in HER2-negative advanced gastric or gastroesophageal junction adenocarcinoma: a randomized, double-blind, phase 3 trial. Nat. Med. 31, 1163–1170. 10.1038/s41591-024-03450-4 39843940

[B27] Shiroiwa T. Fukuda T. Shimozuma K. (2011). Cost-effectiveness analysis of trastuzumab to treat HER2-positive advanced gastric cancer based on the randomised ToGA trial. Br. J. Cancer 105 (9), 1273–1278. 10.1038/bjc.2011.390 21959871 PMC3241558

[B28] Shu Y. Ding Y. Zhang Q. (2022). Cost-effectiveness of nivolumab plus chemotherapy vs. chemotherapy as first-line treatment for advanced gastric cancer/gastroesophageal junction cancer/esophagel adenocarcinoma in China. Front. Oncol. 12, 851522. 10.3389/fonc.2022.851522 35515123 PMC9065445

[B29] Smyth E. C. Nilsson M. Grabsch H. I. van Grieken N. C. Lordick F. (2020). Gastric cancer. Lancet 396 (10251), 635–648. 10.1016/S0140-6736(20)31288-5 32861308

[B30] Spagnolo F. Boutros A. Cecchi F. Croce E. Tanda E. T. Queirolo P. (2021). Treatment beyond progression with anti-PD-1/PD-L1 based regimens in advanced solid tumors: a systematic review. BMC Cancer 21, 425–14. 10.1186/s12885-021-08165-0 33865350 PMC8052683

[B31] Van Cutsem E. Sagaert X. Topal B. Haustermans K. Prenen H. (2016). Gastric cancer. Lancet 388 (10060), 2654–2664. 10.1016/S0140-6736(16)30354-3 27156933

[B32] Wu X. Sun Y. Yang H. Wang J. Lou H. Li D. (2024). Cadonilimab plus platinum-based chemotherapy with or without bevacizumab as first-line treatment for persistent, recurrent, or metastatic cervical cancer (COMPASSION-16): a randomised, double-blind, placebo-controlled phase 3 trial in China. Lancet 404 (10463), 1668–1676. 10.1016/S0140-6736(24)02135-4 39426385

[B33] Xiang Z. Ma L. Fu Y. Pan Y. (2024). Cost-effectiveness analysis of first-line sintilimab plus chemotherapy vs. chemotherapy alone for unresectable advanced or metastatic gastric or gastroesophageal junction cancer in China. Front. Pharmacol. 15, 1411571. 10.3389/fphar.2024.1411571 39295936 PMC11408219

[B34] Xu J. Jiang H. Pan Y. Gu K. Cang S. Han L. (2023). Sintilimab plus chemotherapy for unresectable gastric or gastroesophageal junction cancer: the ORIENT-16 randomized clinical trial. JAMA 330 (21), 2064–2074. 10.1001/jama.2023.19918 38051328 PMC10698618

[B35] Xu L. Chen M. Angell B. Jiang Y. Howard K. Jan S. (2024). Establishing cost-effectiveness threshold in China: a community survey of willingness to pay for a healthylife year. BMJ Glob. Health 9 (1), e013070. 10.1136/bmjgh-2023-013070 PMC10806867 38195152

[B36] Xu L. Long Y. Yao L. Wang H. Ge W. (2024). Updated cost-effectiveness analysis of tislelizumab in combination with chemotherapy for the first-line treatment of advanced gastric cancer or gastroesophageal junction adenocarcinoma. Front. Oncol. 14, 1477722. 10.3389/fonc.2024.1477722 39737400 PMC11682971

[B37] Zhang L. Wang Y. Li Z. Lin D. Liu Y. Zhou L. (2021). Clinicopathological features of tumor mutation burden, epstein-barr virus infection, microsatellite instability and PD-L1 status in Chinese patients with gastric cancer. Diagn Pathol. 16 (1), 38. 10.1186/s13000-021-01099-y 33933102 PMC8088709

[B38] Zhang P. F. Shi X. Q. Li Q. (2023). Nivolumab plus chemotherapy *versus* chemotherapy alone as first-line treatment for advanced gastric, gastroesophageal junction, and esophageal adenocarcinoma: a cost-effectiveness analysis. Cost. Eff. Resour. Alloc. 21 (1), 65. 10.1186/s12962-023-00476-2 37705023 PMC10500934

[B39] Zhang X. Wang J. Wang G. Zhang Y. Fan Q. Lu C. (2025). First-line sugemalimab plus chemotherapy for advanced gastric cancer: the GEMSTONE-303 randomized clinical trial. JAMA 333 (15), 1305–1314. 10.1001/jama.2024.28463 39992668 PMC11851304

[B40] Zheng Z. Song X. Cai H. Zhu H. (2024). Pembrolizumab combined with chemotherapy *versus* placebo combined with chemotherapy for HER2-negative advanced gastric cancer in China: a cost-effectiveness analysis. Expert Rev. Pharmacoecon Outcomes Res. 24 (8), 1017–1025. 10.1080/14737167.2024.2378986 38979829

