# Supplementary material for: Cadonilimab in combination with chemotherapy for HER2-negative advanced gastric or gastroesophageal junction adenocarcinoma: a cost-effectiveness analysis
Source: Front Pharmacol. 2025 Jul 29;16:1646818. doi: 10.3389/fphar.2025.1646818 (PMC12339465; doi:10.3389/fphar.2025.1646818)
Supplement: Supplementary file 1 [file DataSheet1.pdf]

Supplementary Material

1 Supplementary Tables

Supplementary Table 1. CHEERS Checklist (2022)

Topic	No.	Item	Reported?
**Title and abstract**			
Title	1	Identify the study as an economic evaluation and specify the interventions being compared.	Yes
Abstract	2	Provide a structured summary that highlights context, key methods, results, and alternative analyses.	Yes
**Introduction**			
Background and objectives	3	Give the context for the study, the study question, and its practical relevance for decision making in policy or practice.	Yes
**Methods**			
Health economic analysis plan	4	Indicate whether a health economic analysis plan was developed and where available.	Yes
Study population	5	Describe characteristics of the study population (such as age range, demographics, socioeconomic, or clinical characteristics).	Yes
Setting and location	6	Provide relevant contextual information that may influence findings.	Yes
Comparators	7	Describe the interventions or strategies being compared and why chosen.	Yes
Perspective	8	State the perspective(s) adopted by the study and why chosen.	Yes
Time horizon	9	State the time horizon for the study and why appropriate.	Yes
Discount rate	10	Report the discount rate(s) and reason chosen.	Yes
Selection of outcomes	11	Describe what outcomes were used as the measure(s) of benefit(s) and harm(s).	Yes
Measurement of outcomes	12	Describe how outcomes used to capture benefit(s) and harm(s) were measured.	Yes
Valuation of outcomes	13	Describe the population and methods used to measure and value outcomes.	Yes
Measurement and valuation of resources and costs	14	Describe how costs were valued.	Yes
Currency, price date, and conversion	15	Report the dates of the estimated resource quantities and unit costs, plus the currency and year of conversion.	Yes
Rationale and description of model	16	If modelling is used, describe in detail and why used. Report if the model is publicly available and where it can be accessed.	Yes

Topic	No.	Item	Reported?
Analytics and assumptions	17	Describe any methods for analysing or statistically transforming data, any extrapolation methods, and approaches for validating any model used.	Yes
Characterising heterogeneity	18	Describe any methods used for estimating how the results of the study vary for subgroups.	Yes
Characterising distributional effects	19	Describe how impacts are distributed across different individuals or adjustments made to reflect priority populations.	Yes
Characterising uncertainty	20	Describe methods to characterise any sources of uncertainty in the analysis.	Yes
Approach to engagement with patients and others affected by the study	21	Describe any approaches to engage patients or service recipients, the general public, communities, or stakeholders (such as clinicians or payers) in the design of the study.	Not applicable
**Results**			
Study parameters	22	Report all analytic inputs (such as values, ranges, references) including uncertainty or distributional assumptions.	Yes
Summary of main results	23	Report the mean values for the main categories of costs and outcomes of interest and summarise them in the most appropriate overall measure.	Yes
Effect of uncertainty	24	Describe how uncertainty about analytic judgments, inputs, or projections affect findings. Report the effect of choice of discount rate and time horizon, if applicable.	Yes
Effect of engagement with patients and others affected by the study	25	Report on any difference patient/service recipient, general public, community, or stakeholder involvement made to the approach or findings of the study	Not applicable
**Discussion**			
Study findings, limitations, generalisability, and current knowledge	26	Report key findings, limitations, ethical or equity considerations not captured, and how these could affect patients, policy, or practice.	Yes
Other relevant information			
Source of funding	27	Describe how the study was funded and any role of the funder in the identification, design, conduct, and reporting of the analysis	Yes
Conflicts of interest	28	Report authors conflicts of interest according to journal or International Committee of Medical Journal Editors requirements.	Yes

Supplementary Table 2. Summary of the statistical goodness-of-fit for survival curves.

	Exponential	Weibull	Gamma	Generalized gamma	Gompertz	Log-normal	Log-logistic
Overall population							
OS curve of cadonilimab plus chemotherapy arm							
AIC	1278.838	1257.032	1252.402	1242.615	1271.866	1242.326	1248.348
BIC	1282.559	1264.473	1259.843	1253.776	1279.306	1249.767	1255.789
OS curve of chemotherapy arm							
AIC	1451.138	1408.147	1403.645	1403.392	1427.620	1403.193	1401.797
BIC	1454.859	1415.588	1411.086	1414.553	1435.061	1410.633	1409.238
PFS curve of cadonilimab plus chemotherapy arm							
AIC	1241.025	1225.522	1218.515	1200.968	1240.708	1200.672	1205.230
BIC	1244.745	1232.963	1225.956	1212.129	1248.149	1208.113	1212.671
PFS curve of chemotherapy arm							
AIC	1454.455	1412.750	1404.290	1404.941	1443.341	1448.081	1396.027
BIC	1458.176	1420.191	1411.731	1416.102	1450.782	1455.522	1403.468
High PD-L1 expression subgroup							
OS curve of cadonilimab plus chemotherapy arm							
AIC	437.317	435.691	434.751	432.732	438.674	431.282	433.227
BIC	440.070	441.199	440.258	440.993	444.181	436.789	438.735
OS curve of chemotherapy arm							
AIC	681.779	665.771	665.158	667.158	672.134	670.161	665.787
BIC	684.721	671.654	671.042	675.983	678.018	676.045	671.670
PFS curve of cadonilimab plus chemotherapy arm							
AIC	445.060	442.592	440.269	433.941	446.936	432.830	434.581
BIC	447.814	448.099	445.776	442.202	452.444	438.337	440.088
PFS curve of chemotherapy arm							
AIC	667.087	637.043	632.987	634.091	653.793	634.899	634.439
BIC	670.029	642.926	638.871	642.916	659.676	640.782	640.323
Low PD-L1 expression subgroup							
OS curve of cadonilimab plus chemotherapy arm							
AIC	722.939	710.669	708.046	704.179	718.833	702.785	706.075
BIC	725.996	716.781	714.158	713.348	724.945	708.898	712.188
OS curve of chemotherapy arm							
AIC	744.898	720.709	717.940	718.175	731.558	716.427	717.240
BIC	747.888	726.690	723.921	727.146	737.539	722.408	723.221
PFS curve of cadonilimab plus chemotherapy arm							
AIC	677.596	667.377	663.757	659.065	676.347	657.110	658.890
BIC	680.652	673.490	669.869	668.233	682.460	663.223	665.002
PFS curve of chemotherapy arm							
AIC	692.022	680.805	675.738	674.812	692.570	683.711	664.964

BIC	695.012	686.785	681.719	683.784	698.550	689.692	670.945

OS, overall survival; PFS, progression-free survival; PD-L1, programmed death 1 ligand; AIC, Akaike Information Criterion; BIC, Bayesian Information Criterion.

Supplementary Table 3. Second-line treatment after disease progression in scenario 1

Treatment	Rate		Usage and dosage
	Cadonilimab plus chemotherapy group	Chemotherapy group	
Chemotherapy	60.34%	52.90%	Paclitaxel 80mg/m^2 administered intravenously on days 1and 8, every 3 weeks
Immunotherapy	20.11%	24.64%	Cadonilimab 10 mg/kg administered intravenously on day 1, every 3 weeks
Targeted therapy	19.54%	22.46%	Apatinib 850mg administered orally once a daily

2 Supplementary Figures

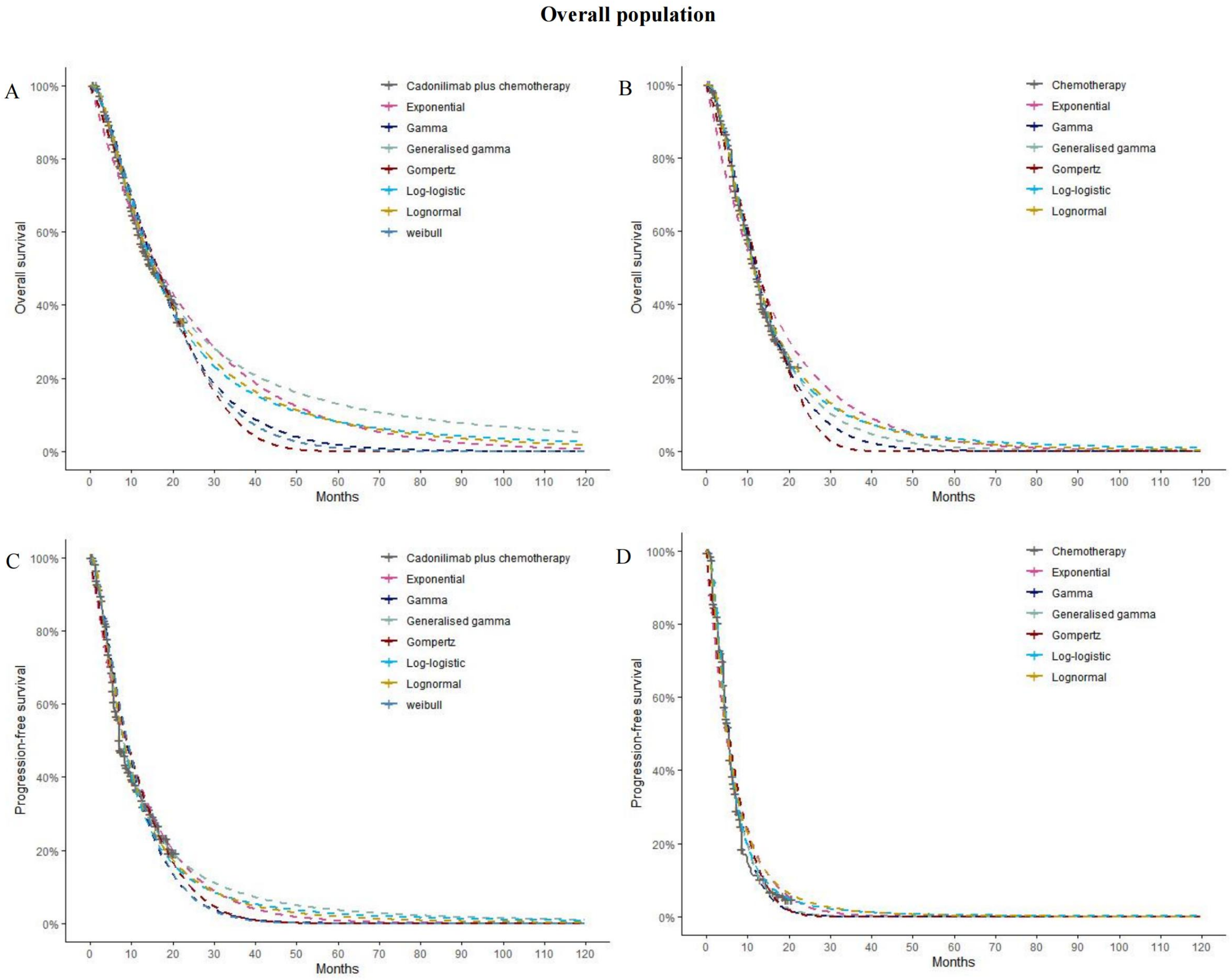

Supplementary Figure 1. Fitting and extrapolation of survival curve in overall population. (A) The results of OS curve in cadonilimab plus chemotherapy arm. (B) The results of OS curve in chemotherapy arm. (C) The results of PFS curve in cadonilimab plus chemotherapy arm. (D) The results of PFS curve in chemotherapy arm. OS, overall survival; PFS, progression-free survival; PD-L1, programmed death ligand 1; CPS, combined positive score.

PD-L1 CPS ≥ 5

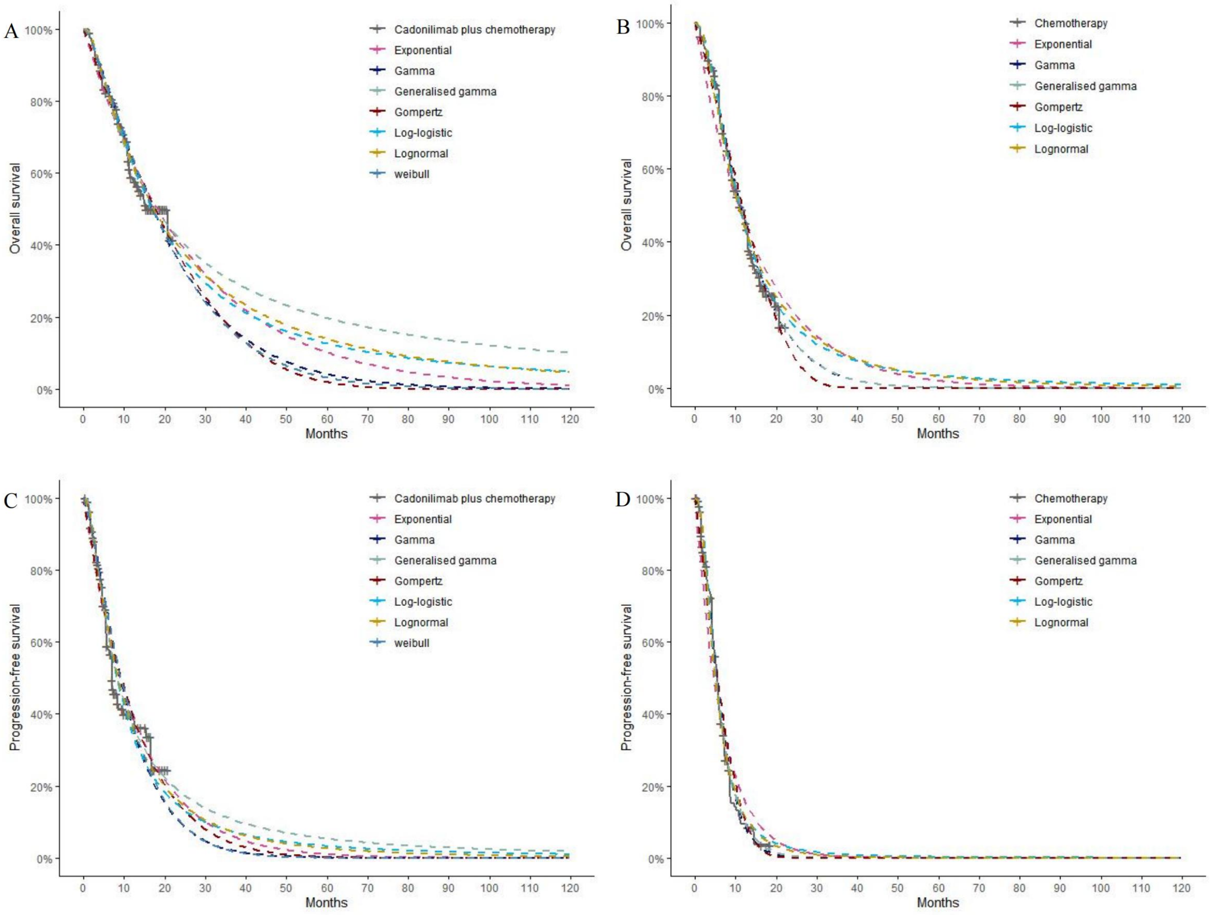

Supplementary Figure 2. Fitting and extrapolation of survival curve for the high PD-L1 expression subgroup. (A) The results of OS curve in cadonilimab plus chemotherapy arm. (B) The results of OS curve in chemotherapy arm. (C) The results of PFS curve in cadonilimab plus chemotherapy arm. (D) The results of PFS curve in chemotherapy arm. OS, overall survival; PFS, progression-free survival; PD-L1, programmed death ligand 1; CPS, combined positive score.

PD-L1 CPS < 5

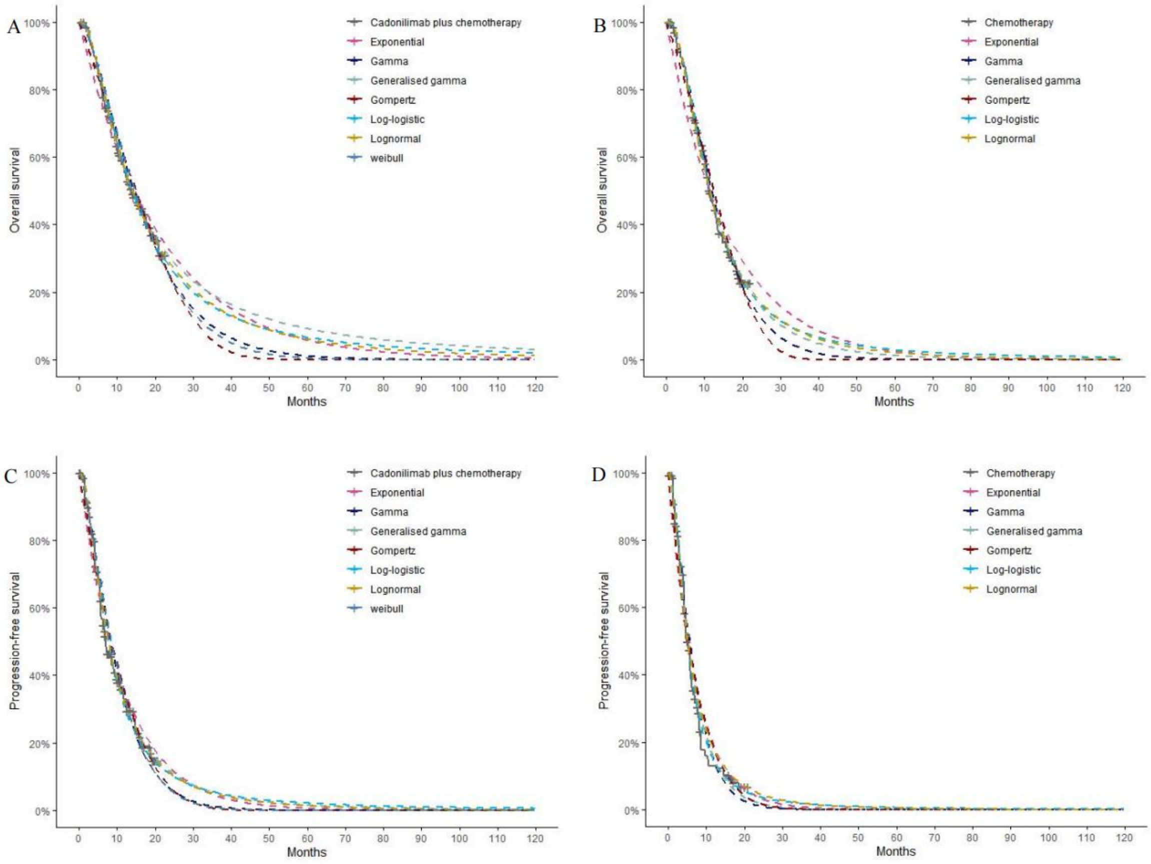

Supplementary Figure 3. Fitting and extrapolation of survival curve for the low PD-L1 expression subgroup. (A) The results of OS curve in cadonilimab plus chemotherapy arm. (B) The results of OS curve in chemotherapy arm. (C) The results of PFS curve in cadonilimab plus chemotherapy arm. (D) The results of PFS curve in chemotherapy arm. OS, overall survival; PFS, progression-free survival; PD-L1, programmed death ligand 1; CPS, combined positive score.
